# Retraction: DNA barcoding identification of Pseudococcidae (Hemiptera: Coccoidea) using the mitochondrial COI gene

**DOI:** 10.1080/23802359.2019.1685160

**Published:** 2019-11-10

We, the Editor and Publisher of *Mitochondrial DNA Part B: Resources,* have retracted the following article:

Sizhu Zheng, Yang Li, Xiaojun Yang, Jingyun Chen, Jing Hua & Yuan Gao (2018). DNA barcoding identification of Pseudococcidae (Hemiptera: Coccoidea) using the mitochondrial COI gene *Mitochondrial DNA Part B: Resources*, 3:1, 419–423 https://doi.org/10.1080/23802359.2018.1457988

This article has been retracted due to concerns with the reliability of the data due to the methodology used, inaccurately described taxonomy, and discrepancies in the number and origin of the specimen sequences reported in the Methods and Table 1. We have not been able to resolve these concerns with the authors and therefore retract this article.

We have been informed in our decision-making by our policy on publishing ethics and integrity and the COPE guidelines on retractions.

The retracted article will remain online to maintain the scholarly record, but it will be digitally watermarked on each page as “Retracted”.

